# Characteristics of recovered COVID-19 patients with recurrent positive RT-PCR findings in Wuhan, China: a retrospective study

**DOI:** 10.1186/s12879-020-05463-z

**Published:** 2020-10-13

**Authors:** Tie-Jun Shui, Chao Li, Hong-bing Liu, Xiaohua Chen, Bi-ke Zhang

**Affiliations:** 1 Yunnan Center for Disease Control and Prevention, Kunming, 650011 Yunnan China; 2 Huangpi District Center for Disease Control and Prevention, Wuhan, 432200 China; 3 Lincang City Center for Disease Control and Prevention, Lincang, 677000 Yunnan China; 4 Beijing Tropical Medicine Research Institute, Beijing Friendship Hospital, Capital Medical University, Beijing, 100050 China; 5 Beijing Key Laboratory for Research on Prevention and Treatment of Tropical Diseases, Capital Medical University, Beijing, 100050 China; 6 Chinese Center for Disease Control and Prevention, Beijing, 102206 China

**Keywords:** SARS-CoV-2, COVID-19, Recurrent, RT-PCR

## Abstract

**Background:**

Two months after the outbreak of coronavirus disease 2019 (COVID-19) in Wuhan, China, tens of thousands of hospitalized patients had recovered, and little is known about the follow-up of the recovered patients.

**Methods:**

The clinical characteristics, reverse transcriptase-polymerase chain reaction (RT-PCR) results from throat swab specimens and the results of serological COVID-19 rapid diagnostic test (RDT) for severe acute respiratory syndrome coronavirus 2 (SARS-CoV-2) were retrospectively reviewed for a total of 758 recovered patients who were previously hospitalized in 17 hospitals and quarantined at 32 rehabilitation stations in Wuhan, China.

**Results:**

In total, 59 patients (7.78%) had recurrent positive findings for COVID-19 on RT-PCR from throat swabs. With regard to antibody detection, 50/59 (84.75%) and 4/59 (6.78%) patients had positive IgG or dual positive IgG/IgM RDT results, respectively.

**Conclusions:**

Some patients who had been quarantined and had subsequently recovered from COVID-19 had recurrent positive RT-PCR results for SARS-CoV-2, and the possibility of transmission of the virus by recovered patients needs further investigation.

**Trial registration:**

Current Controlled Trials ChiCTR2000033580, Jun 6th 2020. Retrospectively registered.

## Background

Coronavirus disease 2019 (COVID-19) is an emerging infectious respiratory disease caused by severe acute respiratory syndrome coronavirus 2 (SARS-CoV-2) that first emerged in early December 2019 in Wuhan, China. As of March 15, COVID-19 had affected 81,062 individuals in China, and 67,041 had recovered after the application of multiple aggressive treatments.

According to the national recommendations for the diagnosis and treatment of pneumonia caused by SARS-CoV-2 (5th edition) and the current status of clinical practice in Hubei Province, real-time reverse transcriptase–polymerase chain reaction (RT-PCR) was used to detect SARS-CoV-2 in respiratory secretions [1, 2]. The patients suspected of having COVID-19 were diagnosed by positive RT-PCR for SARS-CoV-2, and 2 consecutively negative RT-PCR results were used as a criterion for hospital discharge. Then, the patients who had recovered from COVID-19 were quarantined at the rehabilitation stations, and RT-PCR was performed to determine whether they could return to work.

Few studies have described positive RT-PCR test results in patients who have recovered from COVID-19 [3–5], and the clinical characteristics of the recovered COVID-19 patients with recurrent positive RT-PCR results remain unclear. Here, we studied the characteristics of recovered COVID-19 patients with recurrent positive RT-PCR results for SARS-CoV-2 in Huangpi, Wuhan.

## Methods

### Data sources

We conducted a retrospective study focusing on consecutive patients with a confirmed diagnosis of COVID-19 who were hospitalized and quarantined at the rehabilitation stations from February 25, 2020, to March 15, 2020, from Huangpi district in Wuhan, China. The diagnosis of patients was based on the 5th edition of the National Guidelines for the Prevention and Control of the Novel Coronavirus Pneumonia (PC-NCP) published by the National Health Commission of China on February 8, 2020 [1]. The patients with a laboratory-confirmed infection who had 2 consecutive negative RT-PCR results before hospital discharge separated by at least 1 day were enrolled. A total of 758 patients with a confirmed diagnosis of COVID-19 were hospitalized in 17 hospitals and then quarantined at 32 rehabilitation stations in Huangpi district in Wuhan, China. The basic patient information, clinical severity of COVID-19, and results of RT-PCR for SARS-CoV-2 in throat swabs were noted and analysed. This study was approved by the ethics committee of the Yunnan Center for Disease Control and Prevention, Yunnan, China, and all patients gave written informed consent.

### RT-PCR detection of SARS-CoV-2

Real-time RT-PCR was performed on throat swab specimens at Wuhan Ping an Hao Medical Laboratory according to the protocol from DAAN Gene Co., Ltd., of Sun Yat-sen University. SARS-CoV-2 open reading frame 1ab (ORF1ab) and nucleocapsid protein (NP) gene fragments were amplified, and the conditions for amplification were 50 °C for 10 min and 97 °C for 1 min, followed by 40 cycles of 97 °C for 5 s and 58 °C for 30 s. When both targets (ORF1ab and NP) tested positive by specific real-time RT-PCR, the case was considered to be laboratory-confirmed. A cycle threshold value (Ct value) less than 37 was defined as a positive test, and a Ct value more than 40 was defined as a negative test. A medium load, defined as a Ct value from 37 to 40, required confirmation by retesting.

### Serological COVID-19 RDT

Serum was separated by centrifugation at 2500 g for 5 min within 24 h of collection.

The SARS-CoV-2 IgG/IgM RDT Kit from Innovita Biological Technology Co., Ltd. (Beijing, China) was used. Briefly, the assay was performed by adding 10 μl of serum/plasma or 20 μl of whole blood to 2 drops (80 μl) of the assay buffer. Reacting bands were read after 15 min, and the density was determined as either negative or positive. The final results were agreed on by 3 investigators.

### Statistical analysis

All statistical analyses were performed using SPSS version 16.0 (SPSS Inc). Means for continuous variables were compared using independent-group t tests when the data were normally distributed; otherwise, the Mann-Whitney test was used. Binary logistic regression analysis was performed. Probability (p) values less than 0.05 were considered significant.

## Results

### Epidemiological characteristics

As of March 15, 2020, clinical data had been collected from 758 patients with laboratory-confirmed COVID-19 who were quarantined at rehabilitation stations in Huangpi, Wuhan, Hubei Province. Twenty-one (2.77%) of the patients were < 10 years old, 19 (2.51%) were 10–19 years old, 76 (10.02%) were 20–29 years old, 148 (19.53%) were 30–39 years old, 153 (20.18%) were 40–49 years old, 158 (20.84%) were 50–59 years old, 120 (15.83%) were 60–69 years old, 50 (6.60%) were 70–79 years old, and 13 (1.72%) were 80 years old and older. The median age was 48 years (interquartile range 35–58 years), and the mean (SD) age was 46.61 (16.82) years (Table 1). A total of 396 patients (51.40%) were male. All 758 patients had confirmed cases and had had 2 consecutive negative RT-PCR results separated by at least 1 day prior to hospital discharge.

**Table 1:** Clinical characteristics of patients who recovered from coronavirus disease 2019 (COVID-19)

Characteristic	(as of March 15, 2020)	RT-PCR Results								
		Total		Positive		Negative		Difference*	(95% CI)	*P* value
		(n)	(%)	(n)	(%)	(n)	(%)			
		758	100.00%	59	7.78%	699	92.22%			
Age (years)	Minimum to maximum	1–92	/	8–87	/	1–92	/	−3.257 ± 2.279	−7.731 to 1.217	0.1534
	Median (interquartile)	48	(35–58)	52	(35–65)	47	(35–57)			
	Mean ± SD (95% CI)	46.61 ± 16.82	(45.41–47.81)	49.61 ± 18.64	(44.75–54.47)	46.35 ± 16.65	(45.12–47.59)			
	≥80 years	13	1.72%	3	5.09%	10	1.43%			
	70–79 years	50	6.60%	4	6.78%	46	6.58%			
	60–69 years	120	15.83%	13	22.03%	107	15.31%			
	50–59 years	158	20.84%	13	22.03%	145	20.73%			
	40–49 years	153	20.18%	5	8.47%	148	21.17%			
	30–39 years	148	19.53%	10	16.95%	138	19.75%			
	20–29 years	76	10.02%	9	15.25%	67	9.59%			
	10–19 years	19	2.51%	1	1.70%	18	2.58%			
	< 10 years	21	2.77%	1	1.70%	20	2.86%			
Sex	Male	396	51.40%	29	49.15%	367	52.50%	−0.03351 ± 0.06780	−0.1666 to 0.09958	0.62
	Female	362	48.60%	30	50.85%	332	47.50%			
Severity of disease	Mild	229	25.12%	16	27.12%	213	30.47%	0.008608 ± 0.08114	−0.1507 to 0.1679	0.92
	Moderate	465	67.63%	40	67.80%	425	60.80%			
	Severe	60	6.67%	3	5.08%	57	8.16%			
	Critical	4	0.58%	0	0.00%	4	0.57%			

*Difference between (Negative - Positive) ± SEM

Of these patients, 59 (59/758, 7.78%) had positive RT-PCR results when quarantined at rehabilitation stations. Of the 59 patients with recurrent positive RT-PCR results after hospital discharge, one (1.70%) was < 10 years old, 1 (1.70%) was 10–19 years old, 9 (15.25%) were 20–29 years old, 10 (16.95%) were 30–39 years old, 5 (8.47%) were 40–49 years old, 13 (22.03%) were 50–59 years old, 13 (22.03%) were 60–69 years old, 4 (15.83%) were 70–79 years old, and 3 (5.09%) were 80 years old and older. The median age was 52 years (interquartile range 35–65 years), and the mean (SD) age was 49.61 (16.64) years. Twenty-nine patients (49.15%) were male (Table 1).

### Clinical features

The severity of disease ranged from mild to critical among the 758 patients and from mild to severe among the 59 patients with recurrent positive RT-PCR results for SARS-CoV-2. Among the 758 patients, 229 (25.12%) had mild cases, 465 (67.63%) had moderate cases, 60 (6.67%) had severe cases, and 4 (0.58%) had critical cases; the severity was determined according to the guidelines for PC-NCP [1] (Table 1). Of the 59 patients who had recurrent positive RT-PCR results, 16 (27.12%) had mild cases, 40 (67.80%) had moderate cases, 3 (5.08%) had severe cases, and 0 (0.00%) had critical cases (Table 1).

Among all 758 patients, the time interval from the onset of symptoms to the first hospitalization ranged from 1 to 61 days. The median time from the onset of symptoms to the first hospital admission was 8 (3–13) days. The mean (SD) interval was 9.31 (7.78) days from the onset of symptoms to the first hospital admission. Among the 59 patients with recurrent positive RT-PCR results for SARS-CoV-2, the time interval from the onset of symptoms to the first hospitalization ranged from 1 to 33 days. The median time from the onset of symptoms to the first hospital admission was 6 (1–11) days. The mean (SD) interval from the onset of symptoms to the first hospital admission was 7.53 (7.15) days (Table 1). As of March 15, 2020, all patients were asymptomatic, and no more infections had been detected.

### Recurrent positive RT-PCR results for SARS-CoV-2

Of the 59 patients with recurrent positive RT-PCR results for SARS-CoV-2 after hospital discharge, the time from the onset of symptoms to the last positive RT-PCR test result for SARS-CoV-2 ranged from 14 to 61 days. The median time from the onset of symptoms to the first hospital admission was 30 (23–39) days, and the mean (SD) interval was 31.78 (12.17) days. In addition, the time from diagnosis to the last positive RT-PCR test result for SARS-CoV-2 ranged from 13 to 48 days. The median and mean (SD) intervals from the onset of symptoms to the first hospital admission were 24 (20–29) days and 25.53 (7.857) days, respectively. Last, the time from quarantine to the last positive RT-PCR test results ranged from 1 to 19 days. The median and mean (SD) intervals from the onset of symptoms to the first hospital admission were 8 (4–11) days and 7.746 (4.13) days, respectively (Table 2).

**Table 2:** Characteristics of 59 patients who recovered from COVID-19 and had recurrent positive RT-PCR results

	Positive RT-PCR Results			
	From disease onset		From diagnosis	From hospital discharge
	First time	Last time	Last time	Last time
Median (IQR)	1 (−2–7)	30 (23–39)	24 (20–29)	8 (4–11)
Minimum to maximum	−35	14–61	13–48	1/19/2020
Mean ± SD	4.153 ± 7.701	31.78 ± 12.17	25.53 ± 7.857	7.746 ± 4.13
95% CI of the mean	2.146–6.159	28.61–34.95	23.48–27.54	6.67–8.822
Last (weeks) ≥1 week (n)	/	/	/	23
≥2 weeks (n)	/	/	1	33
≥3 weeks (n)	/	11	15	3
≥4 weeks (n)	/	15	25	/
≥5 weeks (n)	/	9	12	/
≥6 weeks (n)	/	15	5	/
≥7 weeks (n)	/	3	3	/
≥8 weeks (n)	/	2	/	/
≥9 weeks (n)	/	4	/	/

Abbreviations: *IQR* interquartile range

**Fig. 1:**
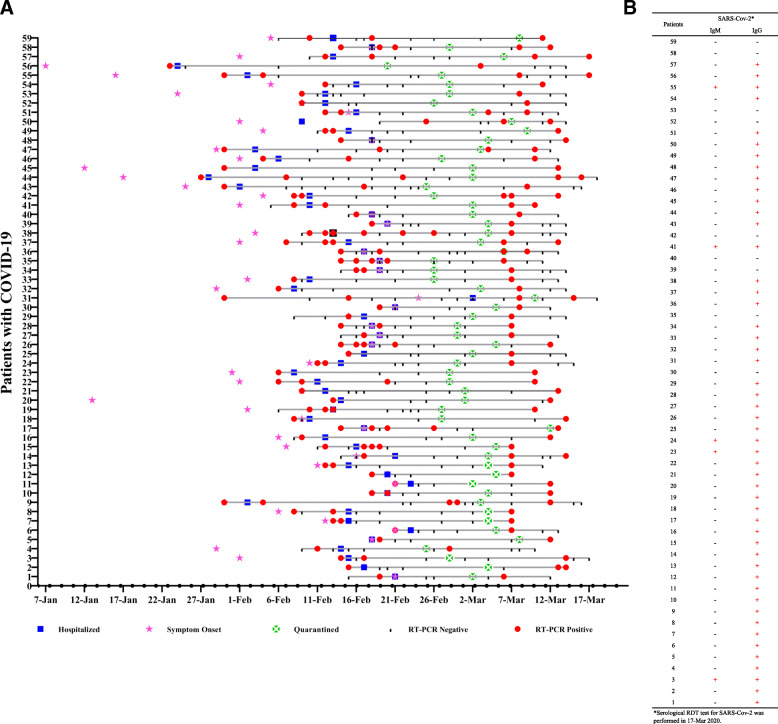
The timeline of recurrent positive RT-PCR findings in patients who had recovered from COVID-19 in Wuhan, China. Details of the timeline (**a**) and serological RDT results (**b**) in recovered COVID-19 patients with recurrent positive RT-PCR findings in Wuhan, China

The timeline of recurrent positive RT-PCR findings in recovered COVID-19 patients in Wuhan, China, is shown in Fig. 1.

### Serological RDTs in patients with recurrent positive RT-PCR results for SARS-CoV-2

The IgG and IgM antibodies for SARS-CoV-2 were detected in the 59 COVID-19 patients who had recurrent positive RT-PCR results for SARS-CoV-2 as of March 17, 2020. Fifty of 59 (84.75%) patients had positive results for the IgG antibody against SARS-CoV-2, while 4 of 59 patients (6.78%) had positive results for both IgM and IgG antibodies against SARS-CoV-2.

The details of the results of the serological RDTs in the patients with recurrent positive RT-PCR results for SARS-CoV-2 are listed in Table 3 and Fig. 1b.

**Table 3:** The serological RDT results for patients with recurrent positive RT-PCR results for SARS-CoV-2

SARS-CoV-2	RT-PCR (n, %)		
	Total	Positive	Negative
Serological RDT	59 (100.00%)	27 (45.76%)	32 (54.24%)
IgM+	5 (8.47%)	3 (11.11%)	2 (6.25%)
IgM-	54 (91.53%)	24 (88.89%)	30 (93.75%)
IgG+	50 (84.75%)	25 (92.59%)	25 (78.12%)
IgG-	9 (15.25%)	2 (7.41%)	7 (21.88%)

### No risk factor identified in the patients with recurrent RT-PCR results for SARS-CoV-2

There were no significant differences in age, sex, disease severity, and time between disease onset and diagnosis between those with and without recurrent positive RT-PCR results. Binary logistic regression analysis showed that age, sex, severity of disease, and time from onset to hospitalization were not risk factors for recurrent positive RT-PCR in quarantined recovered COVID-19 patients. Based on the current data, no risk factor was identified in the patients with recurrent positive RT-PCR results for SARS-CoV-2.

## Discussion

Few previous investigations have evaluated follow-up RT-PCR results for SARS-CoV-2 in patients who have recovered from COVID-19 [3–5]. A few reports have suggested that there are asymptomatic carriers of SARS-CoV-2 who may be able to transmit the virus [6]. Our investigation suggests that among recovered COVID-19 patients, 7.78% (59/758) have recurrent positive RT-PCR results for SARS-CoV-2, with most patients also having positive findings for IgG or IgG/IgM against SARS-CoV-2 on the RDT. These results suggest that recurrent positive RT-PCR results for SARS-CoV-2 commonly appear in patients who have recovered from COVID-19.

Our results show a low prevalence (7.78%; 59/758) of recurrent positive RT-PCR results for SARS-CoV-2 in the throat swab specimens from recovered COVID-19 patients who were quarantined at the rehabilitation stations; these recurrent positive results occurred from 1 to 19 days after quarantine. The results were consistent with a previous study on positive RT-PCR results in patients who had recovered from COVID-19. Four patients with COVID-19 who met the criteria for hospital discharge or the discontinuation of quarantine in China (absence of clinical symptoms and radiological abnormalities and 2 negative RT-PCR results) had positive RT-PCR results 5 to 13 days later [3]. Two other studies also reported that PCR assays turned positive again in 25 of 172 (14.5%) and 15 of 70 (21.4%) discharged patients from Shenzhen [4] and Wuhan [5]. These findings confirmed that a certain proportion of recovered patients may still experience conversion and prolonged nucleic acid positivity regardless of the relief of symptoms and improvements on radiography.

First, RT-PCR has been widely employed in diagnosing viral infections and has yielded few false-positive results [7]. The observed false-negative results have been related to the quality of the kit, the collected sample, or the performance of the test [6]. In this study, specimens were obtained from patients from 17 hospitals who were quarantined at 32 rehabilitation stations, and RT-PCR was performed by trained professionals in a high-quality standardized laboratory. It is less likely that technical reasons were the cause of the recurrent positive RT-PCR results for SARS-CoV-2.

Second, several serological immunoassays have been developed by in vitro diagnostic (IVD) companies for the detection of SARS-CoV-2 viral proteins and antibodies in the serum or plasma. IgM can be detected in patient samples 10 to 30 days after SARS-CoV-2 infection, while IgG can be detected from 20 days onwards [8]. The IgM response occurs earlier than that of IgG, but it then decreases and disappears [9]. In this study, most patients with recurrent positive RT-PCR results (50/59, 84.70%) were positive for IgG against SARS-CoV-2, which implies that the patients had an immune response.

Third, it is presumed that asymptomatic carriers can transmit SARS-CoV-2 [6]. The possibility of transmission by patients who have recovered from COVID-19 and have recurrent positive RT-PCR results for SARS-CoV-2 needs further investigation.

Our study has several limitations. This study had a small sample size of patients with COVID-19. A larger cohort and more detailed follow-up would help elucidate the characteristics of recovered COVID-19 patients with recurrent positive RT-PCR results. In addition, because only basic information, including age, sex, severity of disease, and time from the onset of illness to diagnosis, was collected, no risk factors were found in this study. In the future, more patients need to be enrolled.

## Conclusions

In this study, some recovered COVID-19 patients who had been quarantined had recurrent positive RT-PCR results for SARS-CoV-2. Although there was a low prevalence of recurrent positive RT-PCR results for SARS-CoV-2 in recovered COVID-19 patients, most of them had evidence of an immune reaction, and the possibility of transmission of the virus by these patients needs further investigation.

## Data Availability

The datasets used and/or analysed during the current study are available from the corresponding author on reasonable request.

## Abbreviations

COVID-19: Coronavirus disease 2019
RT-PCR: Reverse transcriptase-polymerase chain reaction
IQR: Interquartile range
PC-NCP: Novel coronavirus pneumonia
RDT: Rapid diagnostic test
